# Glutathione Supplementation Attenuates Oxidative Stress and Improves Vascular Hyporesponsiveness in Experimental Obstructive Jaundice

**DOI:** 10.1155/2015/486148

**Published:** 2015-06-16

**Authors:** Jiaying Chen, Feixiang Wu, Yue Long, Weifeng Yu

**Affiliations:** ^1^Department of Anesthesia & Intensive Care, Eastern Hepatobiliary Surgery Hospital, the Second Military Medical University, Shanghai 200438, China; ^2^Department of Anesthesiology, 81st Hospital of the Chinese PLA, Nanjing 210002, China

## Abstract

We investigated the protective effects and mechanism of glutathione (GSH) on vascular hyporesponsiveness induced by bile duct ligation (BDL) in a rat model. Seventy-two male Sprague-Dawley rats were randomly divided into four groups: a NS group, a GSH group, a BDL + NS group, and a BDL + GSH group. GSH was administrated into rats in the GSH and BDL + GSH groups by gastric gavage. An equal volume of normal saline was, respectively, given in the NS group and BDL + NS group. Blood was gathered for serological determination and thoracic aorta rings were isolated for measurement of isometric tension. Obstructive jaundice led to a significant increase in the serum total bilirubin, AST, and ALT levels. The proinflammatory cytokines levels (TNF-*α* and IL-1*β*), concentration of NO, and oxidative stress markers (MDA and 3-NT) were increased as well. All of those were reduced by the treatment of GSH. Meanwhile, contraction of aorta rings to NA and vasorelaxation to ACh or SNP in the BDL group rats were markedly decreased, while GSH administration reversed this change. Our findings suggested that GSH supplementation attenuated overexpressed ONOO(−) from the reaction of excessive NO with O_2_^∙-^ and protected against obstructive jaundice-induced vascular hyporesponsiveness in rats.

## 1. Introduction

Surgeries in patients with obstructive jaundice (OJ) are associated with high prevalence of postoperative complications and mortality rates [1]. Hemodynamic instability induced by hypotension and impaired vascular reactivity in obstructive jaundice plays a central role in the pathogenesis of the complications in the perioperative period [2]. A decrease in vasoconstrictor tone as well as vascular hyporesponsiveness along with a lesser sensitivity to vasoactive agents such as catecholamine, vasopressin, angiotensin II, and serotonin can ultimately lead to death of patients [3].

Reactive oxygen species (ROS) play a major role in the pathogenesis of cholestasis [4]. ROS contribute to vascular dysfunction and remodeling, an initial episode progressing towards hypertension and atherosclerosis, through oxidative damage by reducing the bioavailability of nitric oxide (NO), impairing endothelium-dependent vasodilatation and endothelial cell growth; hence, cellular events underlying these processes involve changes in vascular smooth muscle cell growth and vasoconstriction [5]. A functional impairment of endothelial cells (ECs) and vascular smooth muscle cells (VSMCs) induced by ROS contributes to endothelial and vascular dysfunction, which probably initiate and induce vascular hyporesponsiveness and vasodilatation. The administration of antioxidants has been shown to exert beneficial effects in the prevention of cholestasis liver injury [6, 7]. However, whether antioxidant therapies maintaining the balance between oxidation and antioxidant systems improve vascular reactivity status remains controversial.

NO is known to play an important role as a key paracrine regulator of vascular tone [8]. Physiologically, NO maintains the health of the vascular endothelium [9]. The enzyme that catalyzes the formation of NO from oxygen is nitric oxide synthase (NOS), which in fact is a whole family of enzymes, including endothelial NOS (eNOS), inducible NOS (iNOS), and neuronal NOS (nNOS). eNOS is the predominant NOS isoform in the vessel wall. Excessive production of NO (nanomolar concentrations) by iNOS hence resulted in an altered contractile response [10]. When large quantities of NO and O_2_^∙−^ collide in the same tissues, they spontaneously interact to form peroxynitrite (ONOO(−)) [11], a potent oxidant, which markedly aggravated oxidative stress (OS) [12].

During OJ, high-concentration bile salts and hyperbilirubinemia may be two major factors contributing to the production of oxygen free radicals, including superoxide anion (O_2_^∙−^), hydroxyl radical, and ONOO(−). Free radicals lead to oxidative damage in many molecules, such as lipids, proteins, and nucleic acids. OJ leads to oxidative injury and inflammation in hepatocytes [13], biliary epithelial and parenchymal cell [14], kidney, heart, intestinal, bladder [15], placenta [16], and red blood cells [17].

GSH, a main nonprotein thiol in cells, serves as a cofactor for a number of antioxidant and detoxifying enzymes [18]. Upon reaction with ROS or electrophiles, GSH becomes oxidized to glutathione disulfide (GSSG), which can be reduced by the GSSG reductase (GR). Thus, the GSH/GSSG ratio reflects the oxidative state and interacts with redox couples to maintain appropriate redox balance [19].

Reaction with GSH was proposed to be a major detoxification pathway of ONOO(−) in the biological system. The redox homeostasis between ONOO(−) and GSH is closely associated with the physiological and pathological processes, for example, vascular tissue prolonged relaxation and smooth muscle preparations, attenuation hepatic necrosis, and activation matrix metalloproteinase-2 [20]. Conversely, the increase in endogenous production of ONOO(−) by inducing a depletion of endogenous glutathione stores aggravates vascular hyporeactivity [21].

Thus, we hypothesized that the elevated concentration of ONOO(−) which is generated by overexpressed NO and O_2_^∙−^ could be critically instrumental in the vascular hyporesponsiveness by increasing oxidative stress during OJ. A supplementation of GSH on the improvement of vascular hyporesponsiveness in rats determines whether GSH administration attenuates ONOO(−) to exert protective effects induced by bile duct ligation.

## 2. Materials and Methods

### 2.1. Animals

A total of 72 pathogen-free, adult male Sprague-Dawley rats (weighing 200–250 g) were obtained from the Shanghai Slac Experimental Animal Centre (Shanghai, China). The rats were housed in individual cages in a temperature-controlled room with alternating 12 h light/dark cycles. Food was withheld 8 h before the start of experiments, but all animals had free access to water. This study was approved by the Animal Care Committee of the Second Military Medical University and performed in accordance with the Guide for the Care and Use of Laboratory Animals.

### 2.2. Experimental Design and Sample Collection

The experimental animals were randomly divided into four groups of 18 rats each: a SHAM-operated group (NS), a bile duct ligated group (BDL + NS), a Sham treated with GSH group (GSH), and a BDL treated with GSH group (BDL + GSH). GSH-treated rats received daily administration of GSH (FuHua Pharmaceutical Co., Ltd., Shanghai, China), 300 mg/kg dissolved in normal saline by gastric gavage for 7 days. In the NS group and BDL + NS group, rats only received an equal volume of normal saline. The dose of GSH given to rats was selected based on information from previous reports [22]. On the eighth day, laparotomy was performed under general anesthesia induced by the injection of chloral hydrate (300 mg/kg, i.p.). The NS and GSH groups were subjected to laparotomy as well as bile duct identification and manipulation without ligation or resection. In the BDL groups, the main bile duct was first ligated using two ligatures approximately 0.5 cm apart and then transected at the midpoint between the two ligatures. GSH administration was then undertaken for another 7 days. At the end of the study, the animals were sacrificed and blood samples were transferred to tubes and immediately centrifuged (3000 r/min for 10 min at 4°C). Serum samples were stored at −20°C in form of frozen for biochemical analyses.

### 2.3. Artery Isolation and Vascular Reactivity Protocol

Thoracic aortas were isolated and prepared for vascular function studies as described previously [23]. Rats were anesthetized using 300 mg/kg chloral hydrate, decapitated, and through opening the abdomen, thoracic aorta was carefully excised and placed in a petri dish filled with cold Krebs solution (KHS) containing (in mM) NaCl 118.5, KCl 4.7, KH_2_PO_4_ 1.2, MgSO_4_ 1.2, NaHCO_3_ 25.0, CaCl_2_ 2.5, and glucose 5.5 at 37°C continuously bubbled with a 95% O_2_-5% CO_2_ mixture (pH 7.4). The aorta was cleaned of excess connective tissue and cut into rings of approximately 3 mm in length. Thoracic aorta segments were mounted on two parallel stainless steel pins for arterial isometric tension recording through a MAP2000 isometric force transducer (Alcott Biotech Co., Ltd., Shanghai, China) connected to a computer. In all experiments, special care was taken to avoid damage to the luminal surface of endothelium. In a subgroup of BDL + NS + Endo(−) group or BDL + GSH + Endo(−) group, the endothelium was mechanically removed by gently rubbing the internal surface with a syringe needle. Segments were suspended in an organ bath containing 20 mL of KHS and subjected to a tension of 2 g which was readjusted every 30 min during a 120 min equilibration period before drug administration.

The vessels were then exposed to KCl (60 mmol/L) to check their functional integrity. After a washout period, isometric contractions were induced by the addition of phenylephrine (PE, 10^−6 ^mol/L). A single concentration of acetylcholine (ACh, 10^−5^ mol/L) was added to the bath in order to assess the endothelial integrity of the preparations after the contraction was stabilized. Endothelium was considered to be intact when this drug elicited a vasorelaxation ≥ 75% of the maximal contraction obtained in vascular rings precontracted with PE. The absence of ACh relaxant action in the vessels indicated the total removal of endothelial cells.

At the end of the equilibration period, dose-response curves for norepinephrine (NA, 10^−9^, 3 × 10^−9^, 10^−8^, 3 × 10^−8^, 10^−7^, 3 × 10^−7^, 10^−6^, 3 × 10^−6^, and 10^−5^ mol/L) in the presence and absence of endothelium were obtained in aortic rings in a cumulative manner. To analyse the participation of NO on the response of NA, the NO synthase inhibitor L-NAME (L-NG-Nitroarginine methyl ester, 10^−4^ mol/L) [24] was added 30 min before the concentration-response curves were performed.

After this, the segments were rinsed several times with KHS over 2 h period, and then cumulative concentration-response curves to ACh (10^−9^, 3 × 10^−9^, 10^−8^, 3 × 10^−8^, 10^−7^, 3 × 10^−7^, and 10^−6^ mol/L), to the NO donor sodium nitroprusside (SNP, 3 × 10^−10^, 10^−9^, 3 × 10^−9^, 10^−8^, 3 × 10^−8^, and 10^−7^ mol/L) were obtained in PE-precontracted segments (PE, 10^−6^ mol/L).

NA responses were expressed as a percentage of the maximum response to KCl. The relaxations induced by ACh or SNP were expressed as a percentage of the initial contraction elicited by PE.

### 2.4. Measurement of TBIL, ALT, and AST in Serum

Rat serum activity of total bilirubin (TBIL), alanine transaminase (ALT), and aspartate transaminase (AST) were measured by an automatic biochemistry analyzer (HITACHI 7110).

### 2.5. Measurement of 3-NT, GSH, MDA, NO, TNF-*α*, and IL-1*β* in Serum

The content of GSH, malondialdehyde (MDA), and NO in serum was detected with reagents kits purchased from Jiancheng Biologic Company (Nanjing, China).

GSH was initiated by the addition of 5,5′-di-thiobis(2-nitrobenzoic acid) and the change in absorbance at 420 nm was monitored by a spectrophotometer.

MDA, the OS product of lipid peroxidation, reacts with thiobarbituric acid under acidic conditions at 95°C to form a pink-colored complex with an absorbance at 532 nm. The results are expressed as nmol or/mL serum.

NO has a half-life of only a few seconds for it is readily oxidized to nitrite (NO_2_^−^) and subsequently to nitrate (NO_3_^−^), which serve as index parameters of NO production. The method for plasma nitrite and nitrate levels was based on the Griess reaction. Total nitrite was measured by spectrophotometry at 545 nm after conversion of nitrate to nitrite by copperized cadmium granules. The results were expressed as *μ*mol/L.

Rat serum 3-nitrotyrosine (3-NT), the OS product of proteins and the proinflammatory cytokines levels (TNF-*α* and IL-1*β*) were measured, using a sandwich enzyme Immunoassay Kit (ELISA) protocol supplied by the manufacturer of the antibodies (Multisciences Biologic Company, Hangzhou, China) and resultant optical density determined, using a microplate reader (Thermo Multiskan MK3) at 450 nm. Results were expressed as pg or ng/mL serum.

### 2.6. Statistical Analysis

Statistical analysis was performed using SPSS version 18.0 software. Data are given as mean ± standard deviation (SD). Analysis of variance (ANOVA) was used to assess differences between multiple groups. A *P* < 0.05 was considered statistically significant.

## 3. Results

### 3.1. General Observations

No deaths were observed during the experiment. Animals that underwent sham surgery (NS group and GSH group) showed no alterations in the clinical conditions. BDL rats were clinically jaundiced within three days. In the BDL rats (BDL + NS group and BDL + GSH group), however, 24 h after surgery the clinical conditions of the animals deteriorated, as shown by decreased activity, irritability, vertical hair, body weight loss, yellowed tails, darkened urine, and pale feces. Seven days after surgery, jaundice was observed in the visceral and parietal peritoneum and varying degrees of ascites, enlarged livers and dilated bile ducts above the obstruction point were also observed in BDL rats. Compared with the rats in the BDL + NS group, rats with supplementation of GSH, demonstrated relatively lighter clinical conditions.

### 3.2. Serum Concentrations of TBIL, ALT, and AST

The serum concentrations of TBIL, ALT, and AST in the BDL + NS group increased visibly compared with those in the NS group (*P* < 0.01, Table 1). The serum concentrations of TBIL, ALT, and AST were significantly lower in the BDL + GSH group than in the BDL group (*P* < 0.01, Table 1).

### 3.3. Levels of GSH, NO, MDA, 3-NT, IL-1*β*, and TNF-*α* in Serum

**Figure 1:**
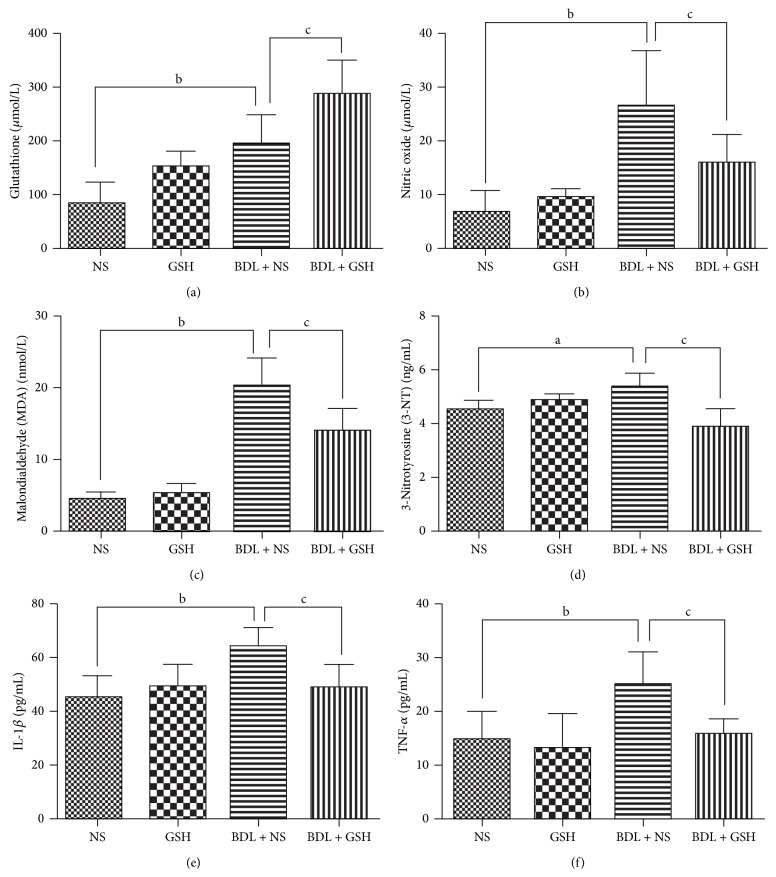
Levels of GSH, NO, MDA, 3-NT, IL-1*β*, and TNF-*α* in serum from the NS group, GSH group, BDL + NS group, and BDL + GSH group (*n* = 6). Data are presented as mean ± SD. ^a^ *P* < 0.05, ^b^ *P* < 0.01 versus NS group; ^c^ *P* < 0.01 versus BDL + NS group.

The levels of GSH, NO, MDA, 3-NT, IL-1*β*, and TNF-*α* were higher in the BDL + NS group than in the NS group (*P* < 0.01, *P* < 0.05, Figure 1). However, GSH was more active in serum and the level of 3-NT, MDA, NO, TNF-*α*, and IL-1*β* was lower in the BDL + GSH group than that in the BDL + NS group (*P* < 0.01, Figure 1).

### 3.4. Vascular Reactivity

**Figure 2:**
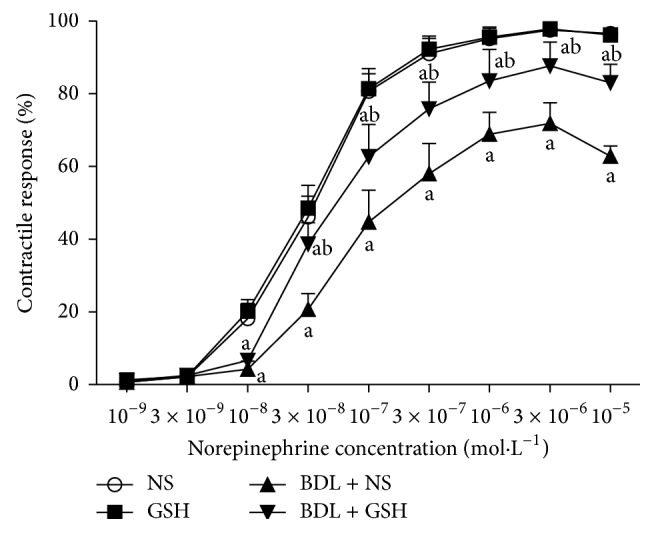
Response elicited by NA in rat thoracic aortic rings from NS group, GSH group, BDL + NS group, and BDL + GSH group (*n* = 6). Data are presented as mean ± SD. ^a^ *P* < 0.01 versus NS group for % maximum response; ^b^ *P* < 0.01 versus BDL + NS group.

Cumulative addition of NA (10^−9^–10^−5 ^mol/L) resulted in concentration dependent contractions in aortas of all groups. The maximum contractile responses to NA (10^−8^–10^−5 ^mol/L) in the aortas from the NS group rats in the presence of endothelium were significantly (*P* < 0.01) greater than the BDL + NS group rats. Compared with the BDL + NS group, GSH pretreatment (BDL + GSH group) enhanced contractile response of thoracic aortic rings to NA (3 × 10^−8^–10^−5^ mol/L) (Figure 2).

**Figure 3:**
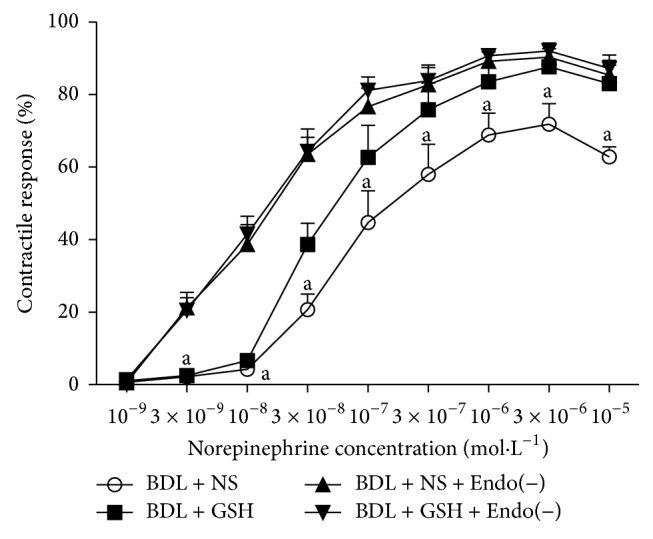
Response elicited by NA in rat thoracic aortic rings from the BDL + NS group, BDL + GSH group, BDL + NS + Endo(−) group, and BDL + GSH + Endo(−) group (*n* = 6). Endothelial was mechanically stripped in BDL + NS + Endo(−) group and BDL + GSH + Endo(−) group. Data are presented as mean ± SD. ^a^ *P* < 0.01 versus BDL + NS + Endo(−) group for % maximum response.

Although endothelium-denuded aortic rings showed a higher contractile response to NA (3 × 10^−9^–10^−5^ mol/L), there were also no significant differences between BDL + GSH and BDL + GSH + Endo(−) (Figure 3), indicating the necessity of endothelium presence for beneficial vascular effect of GSH.

**Figure 4:**
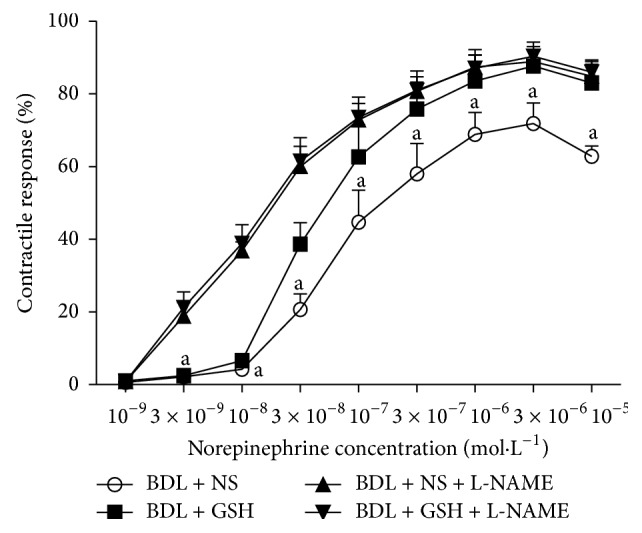
Response elicited by NA in rat thoracic aortic rings from the BDL + NS group, BDL + GSH group, BDL + NS + L-NAME group, and BDL + GSH + L-NAME group (*n* = 6). Incubation of L-NAME in the BDL + NS + L-NAME group and BDL + GSH + L-NAME group. Data are presented as mean ± SD. ^a^ *P* < 0.01 versus BDL + NS + L-NAME group for % maximum response.

Preincubation of aortic rings with L-NAME significantly increased the contractile response of aortic rings from BDL + NS + L-NAME group rats to NA (3 × 10^−8^–10^−5^ mol/L). Likewise, GSH did not modify the contractile response in the BDL + NS + L-NAME and BDL + GSH + L-NAME group (Figure 4).

**Figure 5:**
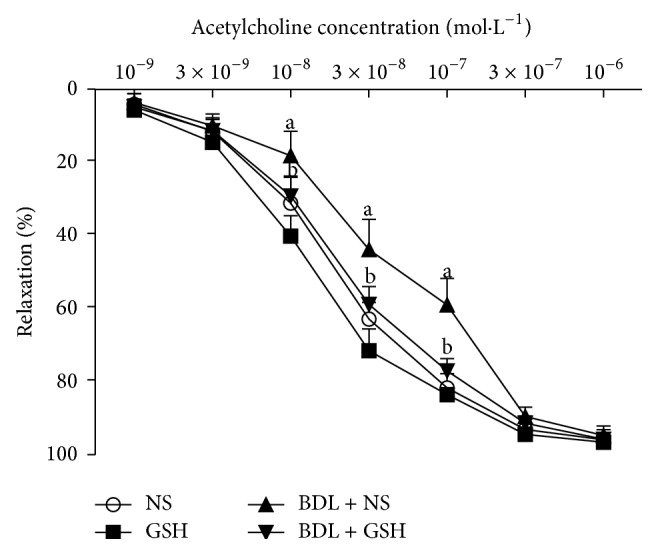
Response elicited by ACh in rat thoracic aortic rings from NS group, GSH group, BDL + NS group, and BDL + GSH group (*n* = 6). Data are presented as mean ± SD. ^a^ *P* < 0.01 versus NS group for % maximum response.

**Figure 6:**
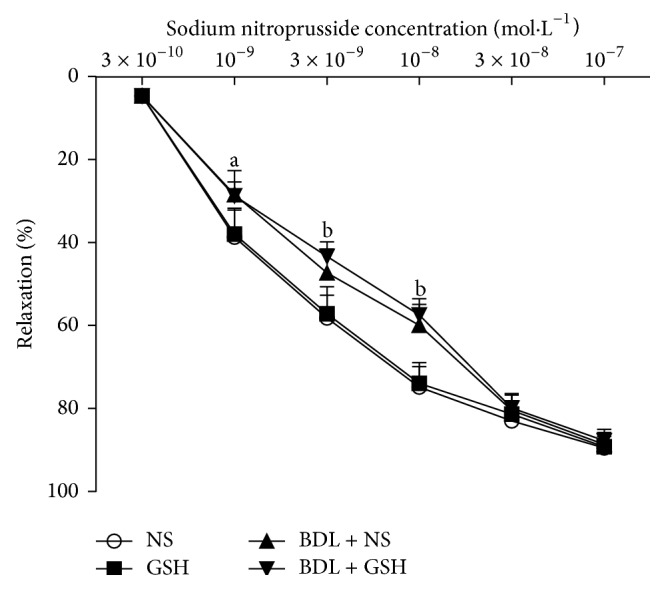
Response elicited by SNP in rat thoracic aortic rings from NS group, GSH group, BDL + NS group, and BDL + GSH group (*n* = 6). Data are presented as mean ± SD. ^a^ *P* < 0.05, ^b^ *P* < 0.01 versus NS group for % maximum response.

Aortic rings precontracted with PE from the BDL + NS group showed a significant reduction in relaxation response to ACh (*P* < 0.01, 10^−8^–10^−7^ mol/L) (Figure 5) and SNP (*P* < 0.01, *P* < 0.05, 10^−9^–10^−8^ mol/L) (Figure 6) as compared to the NS group. The relaxation to ACh (*P* < 0.01, 10^−8^–10^−7^ mol/L) was significantly greater in aortic rings from the BDL + GSH group than in those from the BDL + NS (Figure 5), while the SNP-induced response was similar in the BDL + NS and BDL + GSH groups (Figure 6).

These results suggest that GSH enhances the endothelium dependent vascular responses in the pathogenesis of cholestasis, without affecting the endothelium-independent mechanisms.

## 4. Discussion

The present study demonstrated that oral administration of GSH reduced total bilirubin, ALT, AST, and proinflammatory cytokines levels in the systemic circulation in an experimental OJ animal model with BDL for 7 d. Moreover, GSH supplementation to the rats in the BDL + GSH not only reduced serum 3-NT levels, a protein damage marker induced by ONOO(−), but substantially improved vascular hyporesponsiveness.

### 4.1. Oxidative Stress in Obstructive Jaundice

Biliary obstruction is associated with an intense state of OS affecting both the liver and extrahepatic organs [25]. OJ increases OS, characterized by a rise of systemic MDA and a decrease in cellular antioxidant defenses, such as GSH and antioxidant enzymes [26]. Overproduction of ROS, which take a pivotal role of OS, has been shown to induce hypotension and fluid depletion [27]. Intrahepatic and extrahepatic accumulation of ROS is thought to be an important cause for the possible mechanisms of the pathogenesis of cholestatic tissue injury from jaundice. ROS, produced in the course of several biochemical reactions, are extremely reactive intermediates. These free radicals can cause damage to various biological targets, such as proteins, DNA, and lipids [28].

OJ is greatly assumed to increase hepatic OS, as indicated by elevations in hepatic plasma enzymes and bilirubin fractions. In our study, the levels of TBIL, ALT, and AST as indicatives of hepatic functions were found to increase significantly in OJ, indicating that OJ has affected liver functions. In addition, the increased productions of proinflammatory cytokines, such as TNF-*α* and IL-1*β* are suggested to be responsible for liver damage in OJ. Proinflammatory cytokines exert a considerably amplifying effect in hepatic inflammatory response and cause severe hepatic tissue damage. In our experimental model of OJ, the increased MDA accumulation in serum was indicative of the extent of lipid peroxidation and the level of 3-NT significantly increased by the oxidation damage of ONOO(−).

### 4.2. Overexpressed NO and ONOO(−) in Obstructive Jaundice

ROS are involved in metabolizing NO and, among them, O_2_^∙−^ plays a crucial role since it is source of many other reactive nitrogen intermediates. ONOO(−), a powerful oxidant, is much more reactive than its parent molecules NO and O_2_^∙−^ [29]. Under physiological conditions, the production of ONOO(−) will be low and oxidative damage minimized by endogenous antioxidant defenses [30]. Even modest increases in the simultaneous production of O_2_^∙−^ and NO will greatly stimulate the formation of ONOO(−); a 10-fold increase in O_2_^∙−^ and NO production will increase ONOO(−) formation 100-fold.

Consequently, pathological conditions can greatly increase the production of ONOO(−) [31]. ONOO(−) affects the activity of functional proteins such as receptors, ion-channels, and enzymes by oxidation of cysteine residues (i.e., disulfide-bond formation) and/or nitration of susceptible residues such as tyrosine, tryptophan, and phenylalanine in these proteins [32–36], result in substantial oxidation and potential destruction of host cellular constituents, leading to the dysfunction of critical cellular processes, disruption of cell signaling pathways, and the induction of cell death through both apoptosis and necrosis [31]. It has been shown that ONOO(−) causes tissue damage including adrenergic *α*1-adrenoceptors, arginine vasopressin V1a receptors [37], adrenergic *β*-adrenoceptor [38], pulmonary artery, cardiomyocyte apoptosis, insulin sensitivity [39], and endothelial injury.

Increased level of 3-NT, a marker of oxidative protein modification [40], caused by excessive concentration of ONOO(−), significantly increased the BDL + NS group. Similar results were observed in mesenteric artery from orchidectomized rats in which products generated from NO metabolism, such as ONOO(−) and hydrogen peroxide, are able to induce relaxation [41].

### 4.3. GSH, an Antioxidant in Obstructive Jaundice

GSH, an antioxidant, one of the major drugs used in the treatment of hepatocellular jaundice, prevents damage to important cellular components caused by ROS such as ONOO(−) and peroxides. Panozzo et al. [42] reported that extrahepatic cholestasis reduced bioavailability of blood GSH in rats. The antioxidant defense system is impaired by the decrease in GSH reductase (GR) and in the activity of glutathione peroxidase (GPx) in OJ [43]. Lopze et al. [44] observed that biliary obstruction was accompanied by increased levels of lipid peroxidation in plasma and hepatic tissue and by the depletion of GSH in both biological tissues. Sheen et al. [45] also reported that BDL-induced liver, kidney, and brain tissue damage were associated with increased oxidative stress, represented by decreased total GSH levels in BDL rats. However, Orellana et al. [46] reported that the GSH and MDA levels of kidney and liver tissues increased in the cholestasis-induced rats.

In our study, we found an increase in GSH levels in serum in BDL rats. The content of total serum GSH was increased as a response to the increased oxidative stress in rats. Purucker et al. [47] found that liver GSH significantly increased 24 h (+37%) and 5 days (+53%) after bile-duct ligation. Thereafter, GSH continuously declined at the end of the observation period after 38 days. BDL induced a 3.7-fold increase in hepatic GSH content over 4 days. This increase was not due to the increased hepatic activity of gamma-glutamylcysteine synthetase (GCS); on the contrary, liver GCS activity was substantially diminished to 34% and 11% of normal rats on the 4th and 7th days after ligation, respectively [48]. The level of common bile duct diameter and pressure was on the peak level at 7 days after operation and the excretion of bile to canalicular and sinusoidal was also depressed. Furthermore, the accumulation of GSH entered the systemic circulation and increased the level of GSH in serum on the 7th day in BDL model.

In the present study, we showed that GSH supplementation increased the level of GSH in the BDL + GSH group and reduced serum TBIL, ALT, and AST in OJ rats. Compared with the BDL + NS group, GSH significantly reduced TNF-*α* and IL-1*β* in the BDL + GSH group, which suggested that the protective effects of GSH on liver injury might be mediated by the suppression of the excessive inflammatory response and its cascade induced by OJ. What is more, GSH treatment 300 mg/kg for 14 days attenuated NO, 3-NT, and MDA levels of the serum on the 7 days after ligation, which indicated that GSH reduced the OS induced by OJ. Administration of GSH could exert a significantly unique anti-ONOO(−) effect in BDL + GSH rats and decrease 3-NT content.

Although the antioxidant properties of GSH are thought to play a role in the protective effects, other possible mechanisms, such as modulations of glutathione synthetase (GS), GR, GPx and GSH/GSSG ratio, may be involved. Further researches are needed.

### 4.4. Vasoconstriction and Vasodilator Response in Obstructive Jaundice

In our previous work, we reported that the systolic and diastolic functions of isolated thoracic aorta rings induced by high potassium, NA, PE, and SNP were changed in cholestatic rats. The weakened systolic function might be due to the vascular endothelial dysfunction, vascular smooth muscle cell damage, and decreased expression of *α*1D-AR albumen induced by endotoxemia and hyperbilirubinemia [49]. In this research, we supported the hypothesis that antioxidant supplementation might protect the vascular endothelial dysfunction induced by OS and then improved the vascular hyporesponsiveness to adrenergic agonists. Moreover, we found that rings with endothelium removed from BDL + NS + Endo(−) and BDL + GSH + Endo(−) animals had a greater tension. It enhanced contractility of aortic rings to NA and abolished the protective effect between the BDL + NS group and BDL + GSH group, suggesting that GSH changes at the endothelial level rather than vascular smooth muscle cell.

Endothelial-derived relaxing factors including NO, prostacyclin, and endothelium-derived hyperpolarizing factor released from endothelial cells in vascular vessel lead to relaxation of vascular smooth muscle in an endothelium-dependent manner [50, 51]. Compared with the rings in the BDL + NS group, NA contractility increased with L-NAME incubation in the BDL + NS + L-NAME group and BDL + GSH + L-NAME group, which suggested that the protective effect of GSH was partly due to involvement of NO pathway.

Furthermore, the contribution of NO and endothelium mechanisms in the vasodilator response induced by ACh or SNP were studied. The ACh-induced relaxation response is endothelium-dependent and NO-mediated [52]. The NO donor SNP induces relaxation by direct effect on the smooth muscle and via an endothelium-independent pathway.

Compared with the NS group, we observed a decrease in vascular response to ACh in the BDL + NS group in OJ. Relaxation response to SNP was also significantly attenuated. GSH treatment increased the endothelial-dependent vasodilator response induced by ACh in the BDL + GSH group. Response to SNP was not altered after GSH treatment suggesting that vascular smooth muscle cell was not well protected on the 14th day after administration or the dosage was not enough to alter the vasodilator response.

Bioavailability of endothelial NO alters vascular function, depending on a balance between NO production and degradation. Increased production of NO is beneficial after arterial injury because of its positive effects on vasorelaxation, prevention of platelet aggregation, and regulation of endothelial cell migration [53]. Constitutive NOS (eNOS and nNOS) and not the iNOS, are the isoforms involved in the relaxation.

ACh-induced vasorelaxation is mediated predominantly by eNOS [54]. Increased serum level of NO may be due to increased activity of NOS. However, an increase in iNOS may lead to over production of NO which is an important factor in BDL rats. The iNOS expression has been reported to attenuate the ACh response [55]. Increases in NO level rats may be the first stage of the toxic oxidative reaction that is harmful for the tissues [56]. Indeed, vascular contractility and relaxation may be associated with the deficient basal endothelial activity, besides, increased OS due to excessive production of oxygen-free radicals and decreased antioxidant defense systems may also involve in the process [57]. These results suggest that OJ induces NOS and increases the NO production along with inducing OS.

The effect of GSH on the improvement of endothelial dysfunction could be related to its antioxidant activity. Oxidative degradation of lipids is a well-defined mechanism of cellular damage caused by excessive production of ROS, and MDA is the most widely employed assay used to determine lipid peroxidation. GSH treatment with significantly decreased MDA content indicating that the improvement in endothelium-dependent vasoreactivity from GSH may be partly due to amelioration of lipid peroxidation and oxidative injury in vascular endothelial cells. More specifically, GSH declines ONOO(−) production and increases NO availability. In this case, endothelial dysfunction may be limited to the impairment in the homeostatic balance maintained between GSH and ONOO(−).

### 4.5. Deficiency of This Research

However, the mechanisms of the beneficial effects of GSH on vascular function have not been clarified. We have also questioned the potential anti-inflammatory mechanisms of OJ vasculopathy and among them are the aortic NO and TNF-*α* levels. GSH dose in the current study was selected from previous studies on rats [22]. It should be noted that this dose was much higher than those clinically used in the treatment of hepatocellular jaundice (1200 mg p.o. tid). Additionally, it should be reminded that this is an ex vivo, not an in vivo, study which is performed on the large, but not small, arteries of BDL rats. Therefore, any potential antioxidant activity of GSH requires to be investigated in well-conducted clinical trials performed on humans.

## 5. Conclusion

In conclusion, GSH supplementation attenuates overexpressed ONOO(−) from the reaction of excessive NO with O_2_^∙−^ and protects against OJ-induced vascular hyporesponsiveness in rats. GSH supplementation may be a novel and promising therapeutic strategy for the treatment of OJ-induced vascular hyporesponsiveness during perioperative period. Further researches are necessary to ascertain the possible mechanisms.

## Figures and Tables

**Table 1:** TBIL, ALT, and AST in serum (mean ± SD).

Group	*n*	TBIL *μ*mol/L	ALT U/L	AST U/L
NS	6	0.53 ± 0.24	53 ± 7	95 ± 12
GSH	6	0.37 ± 0.39	49 ± 7	94 ± 9
BDL + NS	6	118.79 ± 9.67^a^	209 ± 22^a^	463.81 ± 49.96^a^
BDL + GSH	6	88.53 ± 22.02^b^	165 ± 29^b^	298.95 ± 35.62^b^

^a^ *P* < 0.01 versus NS group; ^b^ *P* < 0.01 versus BDL + NS group.
